# Right Lower Extremity Osteomyelitis With Alcaligenes faecalis in a Patient With Poorly Controlled Type 2 Diabetes Mellitus

**DOI:** 10.7759/cureus.31172

**Published:** 2022-11-06

**Authors:** John C Smith, Marco Comianos, Wesley Tang, Satish Sarvepalli

**Affiliations:** 1 Division of Internal Medicine, Kettering Health, Dayton, USA; 2 Division of Infectious Disease, Kettering Health, Dayton, USA; 3 Division of Infectious Disease, South Dayton Acute Care Consultants, Dayton, USA

**Keywords:** opportunistic infection, resistance, bacteremia, osteomyelitis, alcaligenes

## Abstract

*Alcaligenes faecalis *(*A. faecalis*) is a Gram-negative, rod-shaped, oxidase(+) and catalase(+), obligate aerobe commonly found in soil or water. It has also been found in human intestinal microbiota and more rarely in hospital settings and is typically associated with opportunistic infections. Although it has intrinsic resistance to many commonly used antibiotics, it is increasingly found to have developed antibiotic resistance. We present a rare case of *A. faecalis* osteomyelitis in a patient with a chronic diabetic foot ulcer.

## Introduction

*Alcaligenes faecalis* (*A. faecalis*) is a Gram-negative, rod shaped, oxidase(+) and catalase(+), obligate aerobe. It is commonly found in soil or water, but has also been found in human intestinal microbiota. In hospital settings, it is most often found in respirators, dialysis machines, or IV solutions, and is associated with opportunistic infections [1,2]. It is generally resistant to aminoglycosides and tetracyclines and susceptible to trimethoprim/sulfamethoxazole (TMP-SMX) and β-lactam antibiotics such as ureidopenicillins, ticarcillin-clavulanic acid, cephalosporins, and carbapenems [3]. However, recent reports have identified genes responsible for intrinsic resistance to β-lactams antibiotics [4]. *A. faecalis* has been associated with a variety of infections including bacteremia, meningitis, endophthalmitis, otitis media, endocarditis, pneumonia, peritonitis, urinary tract infection, and has been previously documented to cause skin and soft tissue infections including diabetic foot ulcers [5,6]. We present a case of *A. faecalis* osteomyelitis of the right lower extremity in a patient with a longstanding diabetic foot ulcer.

## Case presentation

A 55-year-old male with a past medical history of tobacco use disorder, daily marijuana use, reported anaphylaxis to penicillins, and insulin-dependent type 2 diabetes mellitus (most recent hemoglobin A1C 6.3%) presented with worsening pain of a chronic wound of the hallux of the right lower extremity that had been present for eight months. The patient had previously followed with wound care after previous surgical debridement, however, had failed to follow up as scheduled and had not been evaluated or treated with antibiotics in more than four months prior to presenting to the emergency department. The patient had received courses of TMP-SMX, and cefuroxime for other infections within 12 months prior to presenting. 

**Figure 1:**
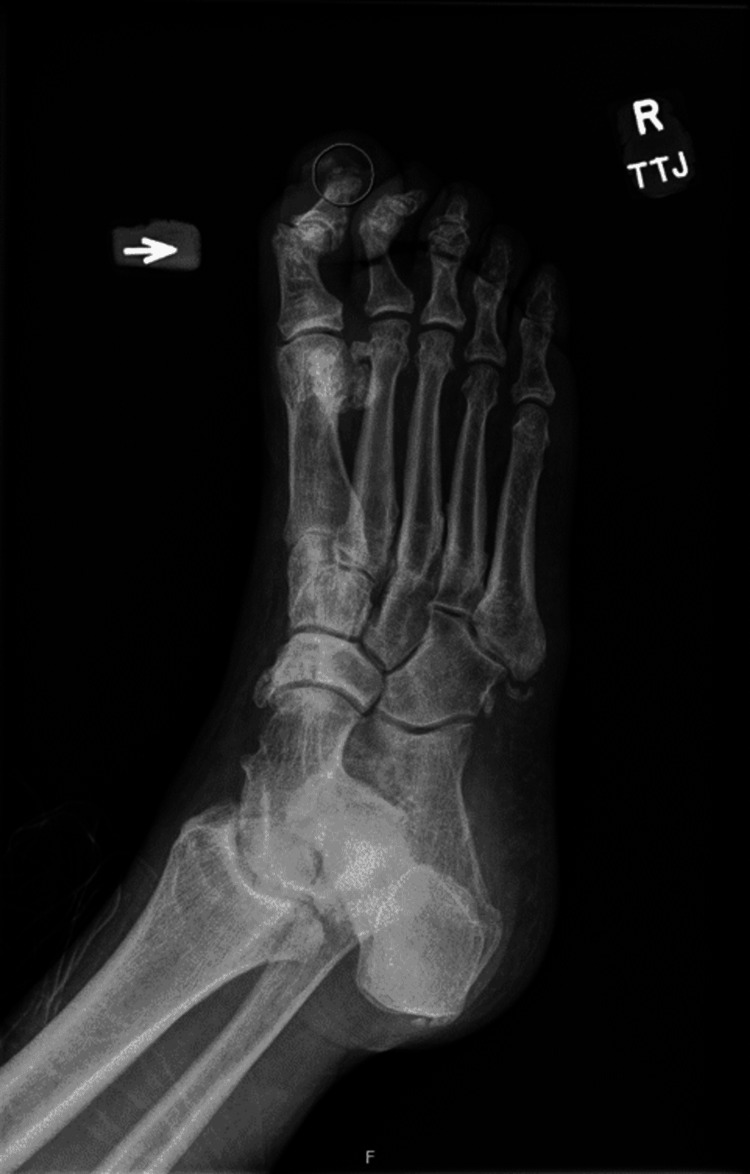
X-ray of right foot demonstrating soft tissue ulceration of the first digit with fragmentation at the tip of the first distal phalanx, concerning for acute osteomyelitis

On initial presentation, the patient was hemodynamically stable and afebrile. Physical exam was significant for a full-thickness ulcerative defect in the distal tuft of the right hallux measuring 1.6 x 1.8 x 0.9 cm with seropurulent exudate with erythema and edema involving the entire right hallux. There was no leukocytosis; however, inflammatory markers, C-reactive protein, and erythrocyte sedimentation rate were elevated. X-ray of the right foot revealed soft tissue ulceration of the first digit with fragmentation at the tip of the first distal phalanx, concerning for acute osteomyelitis (Figure 1). The patient received a single dose of IV metronidazole in the emergency department and was subsequently admitted for further evaluation and treatment. MRI demonstrated findings consistent with osteomyelitis involving the distal tip of the first distal phalanx. Podiatry was consulted for surgical debridement and the patient subsequently underwent sequestrectomy of the bone of the distal phalanx of the toe with fillet flap closure with apparent source control. Post-surgical intervention, with new onset of signs concerning for sepsis, the patient was initiated on empiric antibiotics pending intraoperative cultures with vancomycin and meropenem. The postoperative course was uneventful and the patient remained afebrile without leukocytosis. Intraoperative wound cultures grew *Streptococcus pyogenes*. Sensitivity for the *Streptococcus pyogenes* was obtained showing sensitivity to amoxicillin/clavulanic acid. In the setting of clinical improvement and with no signs of systemic infection and no further organisms isolated, the decision was made to discharge the patient on postoperative day three with oral amoxicillin/clavulanic acid which, after careful review of the medical record, it was found he had tolerated without issue previously. The patient was instructed to complete a 14-day antibiotic course and follow up with podiatry and his primary care physician (PCP).

**Figure 2:**
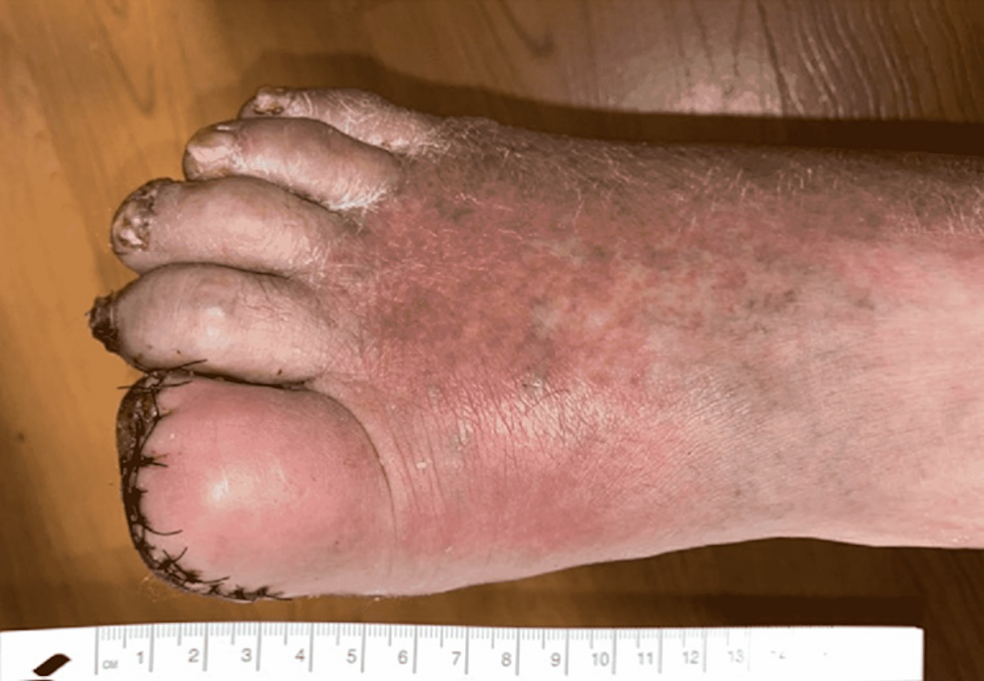
Postoperative findings to the right foot with sutures in place and significant erythema and warmth

Following discharge, the patient subsequently returned to the emergency department less than 24 hours later with complaints of worsening pain as well as increased erythema in the right lower extremity (Figure 2) and was found to have mild leukocytosis (white blood cell count 12.3) for the first time since his initial presentation prior to operative sequestrectomy. The patient was readmitted and antibiotics were switched to cefepime and vancomycin. Intraoperative culture from the initial hospitalization subsequently resulted with* Alcaligenes faecalis* on postoperative day four, identified by matrix-assisted laser desorption/ionization with time of flight and mass spectrometry (MALDI-TOF MS), consistent with previous Gram stain features. The Infectious Disease service was consulted and the patient was switched to oral amoxicillin/clavulanic acid with oral TMP-SMX after the cultured *Alcaligenes faecalis* was found to be sensitive to the latter (Table 1). Of note, the isolated* A. faecalis* had only intermediate sensitivity to piperacillin/tazobactam. Following clinical improvement and resolution of leukocytosis on this antibiotic regimen, the patient was discharged with appropriate follow-up. Approximately two weeks following discharge, the patient remained without systemic signs of infection on an office visit with his PCP.

**Table 1:** Sensitivity of isolated A. faecalis culture to various antibiotics S: Susceptible; I: Intermediate; MIC: Minimum inhibitory concentration

Antibiotic	Interpretation	MIC (µg/mL)
Ciprofloxacin	S	1
Gentamicin	S	4
Levofloxacin	S	1
Piperacillin + Tazobactam	I	64
Tobramycin	S	2
Trimethoprim + Sulfamethoxazole	S	<=20

## Discussion

*A. faecalis* bacteria is usually considered non-pathogenic and has even been proposed as a potential probiotic [7]. Opportunistic infections due to *A. feacalis* do occur in humans [8]; however, previous cases of *A. faecalis* osteomyelitis have only been rarely reported. Skin and soft tissue infections of *A. faecalis*, including infections associated with diabetic foot ulcers, have been previously described; however, to the authors’ knowledge, only three other cases of *A. faecalis *osteomyelitis have been reported, all three in patients with diabetic foot ulcers [5,6]. 

*A. faecalis* is typically associated with opportunistic infections; however, it has also been associated with significant antimicrobial resistance. Organisms that are resistant to all commercially available antimicrobials are considered pandrug-resistant (PDR). Although unusual, PDR bloodstream infections of *A. faecalis* species have been recently reported [9]. In the present case, the patient experienced an acute clinical worsening after being transitioned from broad-spectrum empiric antibiotics to a more focused antimicrobial regime of amoxicillin/clavulanic acid to which his Streptococcus pyogenes infection was shown to be sensitive. After adding TMP-SMX, an antibiotic to which this particular *A. faecalis *isolate was susceptible, he clinically improved and continued to do well several weeks following discharge. Unfortunately, data for the A. faecalis sensitivity to amoxicillin/clavulanic acid was not obtained. 

The literature shows only a few cases of *A. faecalis *osteomyelitis, which have all occurred in cases of diabetic foot ulcer infection. Of the reported cases of *A. faecalis *osteomyelitis, one was sensitive to all tested antibiotics while the other had only intermediate sensitivity to piperacillin/tazobactam and amoxicillin/clavulanic acid and was deemed to have extensive drug resistance by the authors [6]. More generally, previously documented cases of *A. faecalis* have been successfully treated with amoxicillin/clavulanic acid [5,10]; however, more recently a case of a patient with extended-spectrum β-lactamase (ESBL)-producing *A. faecalis* infection has been reported [11]. In a sample of 188 patients with chronic suppurative otitis media, *A. faecalis* was always susceptible to aminoglycosides amikacin, cephalosporins, and colistin and usually susceptible to gentamicin, tobramycin, and piperacillin/tazobactam (90% susceptibility), with good susceptibility (75%) to TMP-SMX [12]. However, a more recent study of 61 patients showed the best sensitivity rate to* A. faecalis* was 66.7% for three antibiotics (imipenem, meropenem, and ceftazidime) with two antibiotics (ciprofloxacin and piperacillin/tazobactam) sensitivity rates less than 50% [2]. A recent case study of cavitary pneumonia reported complete resistance to all antibiotics available commercially [13]. Recent empiric treatment recommendations for *A. faecalis* include the use of piperacillin-tazobactam, TMP-SMX, meropenem, and ceftazidime with minocycline as an alternative treatment option [14].

## Conclusions

Diabetic foot ulcers with *A. faecalis* infection have been occasionally reported in the literature; however, *A. faecalis *has been only rarely associated with osteomyelitis. We have presented another case of *A. faecalis* associated osteomyelitis with antibiotic resistance beyond what has been previously reported as typical for *A. faecalis*. Antibiotic resistance has been highlighted in the recent literature regarding *A. faecalis*. In this setting, optimal treatment of diabetic foot ulcers is important in minimizing comorbidities and the need for more invasive interventions including limb amputation and/or antimicrobials; when required, definitive antibiotic therapy for *A. faecalis* should consider the propensity for antimicrobial resistance in *A. faecalis* and should be promptly initiated based on culture results and susceptibility data.
